# The System Research of the Molecular Mechanism of Quyushengxin Capsule in the Treatment of Osteonecrosis of the Femoral Head

**DOI:** 10.1155/2022/2968075

**Published:** 2022-01-11

**Authors:** Xia Du, Lintao Zhao, Yuan Qiao, Yuan Liu, Dong Guo

**Affiliations:** ^1^Institute of Traditional Chinese Medicine, Shaanxi Academy of Traditional Chinese Medicine, Xi'an, Shaanxi 710003, China; ^2^Center for Post-Doctoral Studies, China Academy of Chinese Medical Sciences, Beijing 100700, China

## Abstract

Osteonecrosis of the femoral head (ONFH) is a chronic and irreversible disease that has a risk of eventually developing into a joint collapse and resulting in joint dysfunction. Quyushengxin capsule (QYSXC) is an effective and safe traditional Chinese medicine used in the treatment of ONFH. In this present study, an integrated approach was used to investigate the mechanism of QYSXC in the treatment of ONFH, which contained systems pharmacology, molecular docking, and chip experiment. In the systems pharmacology, target fishing, protein-protein interaction (PPI), Kyoto encyclopedia of genes and genomes (KEGG) pathway enrichment analysis, and herbs-compounds-targets-pathways (H-C-T-P) network construction were performed to study the mechanism of QYSXC in the treatment of ONFH. The results showed that 15 key compounds, 8 key targets, and 8 key signaling pathways were found for QYSXC in the treatment with ONFH. Then, molecular docking was performed to further explore the interaction between some key compounds and key targets. After that, the chip experiment was performed to verify some target factors, including ICAM-1, IL-6, IL-1*α*, IL-1*β*, IL-2, IL-4, IL-10, and TNF-*α*. The results of this work may provide a theoretical basis for further research on the molecular mechanism of QYSXC in the treatment of ONFH.

## 1. Introduction

Osteonecrosis of the femoral head (ONFH) is a chronic and irreversible disease that has a risk of eventually developing into a joint collapse and resulting in joint dysfunction [1] and is also a common disabling complication among convalescent severe acute respiratory syndrome (SARS) patients who received corticosteroid therapy [2]. Studies have shown that 80% of patients with ONFH could develop femoral head collapse after 1 to 4 years without effective treatment. In this case, 87% of patients will be performed an artificial hip replacement [3]. At present, the cause and mechanism of ONFH are still under investigation.

Chemical drugs, such as mobike, fenbid, and other anti-inflammatory drugs, clofibrate, lovastatin, and other lipid scavenger drugs, were used in the early clinical treatment of ONFH [4]. Although these drugs have a certain effect in short-term use, the overall treatment effect of them is not ideal. The surgery is the main treatment in the advanced treatment of ONFH, such as core decompression, osteotomy, and bone cement filling technique. However, it is difficult to control the long-term effects of various femoral head preservation operations. At present, hip arthroplasty is one of the most common treatments for ONFH. Nonetheless, the artificial joint prosthesis has a service life, and the hip revision is more difficult, riskier, and with more complications. In recent years, traditional Chinese medicine (TCM) has increasingly shown its advantages in the treatment with ONFH, and it can effectively improve the symptoms of the patients, control the development of the disease, and improve joint function and the life quality of the patients as well.

ONFH belongs to the category of “bone arthralgia” and “bone erosion” in Chinese medicine. Quyushengxin capsule (QYSXC) is a TCM based on the theory of etiology and pathogenesis of TCM and formed in long-term clinical practice. It is composed of Astragali radix (Huangqi), Cinnamomi ramulus (Guizhi), Sparganii rhizoma (Sanleng), Curcumae rhizoma (Ezhu), Bolbostemmatis rhizoma (Beimu), and Strychni semen (Maqianzi). Clinical studies have shown that QYSXC was able to improve the overall treatment efficiency and improve the clinical indicators with ONFH patients [5]. In basic research, the main chemical components and pharmacological effects of the herbs constituting the formula have been preliminarily clarified. For example, Sparganii rhizoma is commonly used drugs for promoting blood circulation and removing blood stasis with antithrombotic, anti-inflammatory, and antifibrous tissue hyperplasia effects, which mainly contains saponins and flavonoids [6]. Strychni semen mainly contains alkaloids, terpenes, and organic acid compounds, and its main pharmacological effects are activating meridians to stop pain and eliminating stagnation and swelling [7]. Astragali radix, cinnamomi ramulus, curcumae rhizoma, and bolbostemmatis rhizoma have also been reported to have anti-inflammatory and immune regulating effects [8–11]. In addition, the pharmacodynamic and acute toxicity experiments have also proved that QYSXC functioned well of being anti-inflammatory and analgesic and less toxic [12]. However, there are few studies on the pharmacodynamic material basis and mechanism of QYSXC in the treatment of ONFH, and its multicomponent and multitarget mechanism is still unclear.

In the present study, an integrated approach was adopted to investigate the mechanism of QYSXC in the treatment of ONFH, which contained systems pharmacology, molecular docking, and chip experiment. Firstly, target fishing, protein-protein interaction (PPI), Kyoto encyclopedia of genes and genomes KEGG pathway enrichment analysis, and herbs-compounds-targets-pathways (H-C-T-P) network construction were performed to thoroughly analyze the mechanism of QYSXC in the treatment of ONFH during the systems pharmacology; then, molecular docking was carried out to further explore the interaction between some key compounds and key targets; finally, the chip experiment was performed to verify some target factors. In short, the results showed that QYSXC was able to treat ONFH through multiple components, multiple targets, and multiple pathways. This work may provide a theoretical basis for further research on the molecular mechanism of QYSXC in the treatment of ONFH.

## 2. Materials and Methods

### 2.1. Systems Pharmacology

#### 2.1.1. Collection of Chemical Components for QYSXC

The chemical components contained in the herbs were collected by Traditional Chinese Medicine Systems Pharmacology (TCMSP) database and analysis platform (https://tcmsp-e.com/) [13], National Center for Biotechnology Information (NCBI), https://www.ncbi.nlm.nih.gov/), and the Encyclopedia of Traditional Chinese Medicine (ETCM), http://www.tcmip.cn/ETCM/index.php/Home/) [14] traditional Chinese medicine data platforms for QYSXC.

#### 2.1.2. ADME Virtual Screening of Potential Medicinal Ingredients

The computer ADME virtual screening was performed to obtain the potential active ingredients with good ADME properties. The pre-OB and pre-Caco2 prediction models were selected to calculate the OB and Caco-2 values [15–17]. The OBioavail 1.1 model used partial least squares method, multiple linear regression, and support vector machine for mathematical modeling, and the drug metabolism and transport can be simulated in the body through cytochrome P450 3A4 and P-glycoprotein, and then it determined the threshold of OB (OB ≥ 30%) by using the 5-fold cross validation and independent external validation. A strong robust prediction model built with 100 drugs was used in the pre-Caco2 model to predict the drug absorption rate in the small intestinal epithelial cells, namely, Caco-2 values, and the threshold of Caco-2 is -0.40.

#### 2.1.3. Target Fishing

An integration of various approaches was used to predict the most likely protein targets of the potential active compounds, which was proposed by Yu et al. [18]. Firstly, they have developed a systems drug targeting (SysDT) model on the basis of random forest (RF) and support vector machine (SVM), which is incorporated a large scale of chemical, genomic, and pharmacological data. The training set of this model contained 6511 drugs and 3999 proteins. The probability of interactions between each molecule and 3,999 targets was obtained according to the model. The top 30 high-ranking proteins in both RF and SVM models were extracted, and the overlap targets between them were selected as candidate targets; secondly, a chemical fingerprint similarity was employed based on similarity ensemble approach (SEA), https://sea.bkslab.org/) [19]; finally, data mining was conducted to extract the information involved in the molecular targets, which can be taken as a complementary method for target fishing. All targets were converted by UniProt database (https://www.uniprot.org/) and corrected to the official shorthand.

#### 2.1.4. Target Genes Linked to Disease

All obtained targets were mapped to the diseases in therapeutic target database (TTD), http://db.idrblab.net/ttd/) [20], comparative toxicogenomics database (CTD), https://ctdbase.org/) [21], and pharmacogenetics and pharmacogenomics knowledge base (PharmGKB), https://www.pharmgkb.org/) [22] databases. Notably, ONFH is a pathological evolution process involving many diseases, such as osteoarthritis, inflammation, pain and ischemia reperfusion injury, lipid metabolism disorder, osteoporosis, intravascular coagulation, and villonodular synovitis. In this present study, the pharmacological action characteristics of the herbs constituting QYSXC and its clinical application were comprehensively considered. Its main functions are promoting blood circulation and removing blood stasis, and anti-inflammatory and analgesic effects. Hence, osteoarthritis, inflammation, pain, and ischemia reperfusion injury were selected as the diseases related to ONFH. After that, we combined the results of 3 databases and removed duplicate genes to get the target genes corresponding to the ONFH for QYSXC.

#### 2.1.5. Construction of Protein-Protein Interaction (PPI) Network

The target genes obtained by the above screening were imported in STRING 11.0 (https://string-db.org/) [23] to construct the protein-protein interaction (PPI) network. The protein type was set to “Homo sapiens,” the minimum interaction threshold is set to “medium confidence (>0.400),” and other parameters are set as default. After that, the results were transferred to Cytoscape 3.8.0 software for constructing the PPI network.

#### 2.1.6. KEGG Pathway Enrichment Analysis

The KEGG pathway enrichment analysis was performed by using clusterProfiler package of program R, and the results were visualized by program R.

#### 2.1.7. Network Construction and Analysis

The H-C-T-P network was constructed by Cytoscape 3.8.0 software [24], and the topological properties, such as degree centrality (DC), betweenness centrality (BC), and closeness centrality (CC), were analyzed by the Network Analyzer plugin in the software.

### 2.2. Molecular Docking

Molecular docking was carried out to investigate the interactions between some key components and targets. The structures of the key components were obtained from the PubChem database (https://pubchem.ncbi.nlm.nih.gov). The structures of the targets were obtained from the PDB database (http://www.pdb.org). The docking simulation was conducted with AutoDock 4.2.6 software [25]. During the docking calculations, hydrogen atoms and Gasteiger charges were added to the protein by using the automated docking tool. The auxiliary program Autogrid was used to set the docking boxes, which were defined according to the crystal structures of complex of the proteins with known ligands. The Lamarckian genetic algorithm (LGA) was adopted for each docking progress.

### 2.3. Experimental Validation

#### 2.3.1. Animals

Sprague-Dawley (SD) rats weighing 180–220 g (half male and female) were purchased from the Experimental Animal Facilities of Xi'an Jiaotong University. The certificate number was SCXK2012-003. The rats were fed with a standard pellet diet and tap water at random, and they were housed and maintained in 12 h light/darkness with standard humidity and temperature in the laboratory. The principles of laboratory animal care guidelines, approved by the Animal Ethics Committee at the Shaanxi academy of traditional Chinese medicine, have been strictly observed. All efforts were made to minimize animal suffering and reduce the animals used for experiments.

#### 2.3.2. Reagents and Materials

The drug of QYSXC was produced by Shaanxi academy of traditional Chinese medicine (lot number: 20181213). Xianlinggubao Capsule (XLGBC) was purchased from Sinopharm Group Tongjitang (Guizhou) Pharmaceutical Co., Ltd. (lot number: 180503). LPS was purchased from Sigma-Aldrich (St. Louis, MO, USA). Methylprednisolone was purchased from Pfizer (New York, NY, USA).

#### 2.3.3. Drug Administration and Grouping

The interior powder (0.3 g/capsule) after being removed from the shells of QYSXC was blended with deionized water as a working mixture for use. The doses selected were subject to the conversion of clinical adult dosages. According to the instructions, the clinical adult dose was four capsules for once, three times a day. After acclimatization for a period of three days, they were randomized into 6 groups: blank control group, model group, QYSXC high dose group (0.48 g/kg), QYSXC medium dose group (0.24 g/kg), QYSXC low dose group (0.12 g/kg), and XLGBC positive control group (0.4 g/kg). Model group received two doses of intraperitoneal injection of lipopolysaccharide (20 *μ*g/kg) at an interval of 24 hours; 24 hours later, the rats received three doses of intramuscular injection of methylprednisolone (40 mg/kg) at an interval of 24 hours. After 8 weeks, QYSXC and XLGBC groups were administered by intragastrically (i.g.) daily for 4 weeks according to the above specified dose. The control group and model group were given by an equal volume of deionized water in parallel.

#### 2.3.4. Cytokine Assays

By means of abdominal aortic method and under general anaesthesia, the blood sample was collected from each group after 4-week treatment. Blood samples were centrifuged to isolate the serum, which was stored at −80°C. These cytokine analyses were performed in accordance with the manufacturer's instructions for the xMAP technology with multiplex beads. In addition, these plates were measured using the Bio-Plex MagPix System and analyzed with the Bio-Plex Manager version 6.1 (Luminex, Austin, TX, USA), with the agent being Wayen Biotechnologies (Shanghai, China), Inc. The Bio-Plex software calculated cytokine concentrations on the basis of fluorescence values derived from a recombinant cytokine standard included in the 96-well plate, and the software then generated a separate standard concentration curve for each of the 8 cytokines sampled. Each standard curve was created by means of eight concentration points. A curve fitted by a five-parameter logistic equation was generated by a nonlinear least-squares minimization algorithm, and the high and low limits of detection were determined. None of the cytokine levels were above or below the detection range. The following cytokines GM-CSF, IL-1*α*, IL-1*β*, IL-2, IL-4, IL-6, IL-10, ICAM-1, TNF-*α*, and VEGF were measured. Results are expressed as picograms per milliliter.

#### 2.3.5. Statistics Analysis

The heatmap was drawn by Me V software. All data were expressed as the mean ± standard deviation (SD). Graphpad Prism 5.01 software was used to ascertain statistically significant differences. The levels of significance were determined using a one-way analysis of variance (ANOVA) followed by Dunnett's multiple comparison test. *P* < 0.05 was considered statistically significant, and *P* < 0.01 was considered to have an extremely significant difference.

## 3. Results and Discussion

### 3.1. The Chemical Components of QYSXC

A total of 551 compounds were obtained by means of TCMSP, NCBI, and ETCM databases, including 87 ingredients in astragali radix, 248 ingredients in cinnamomi ramulus, 30 ingredients in sparganii rhizoma, 81 ingredients in curcumae rhizoma, 43 ingredients in bolbostemmatis rhizoma, 62 ingredients in strychni semen. These compounds are mainly organic acid ester compounds, saponin compounds, flavonoid compounds, and phenolic compounds.

### 3.2. Screening Results of the Potential Active Compounds

In this paper, the pre-OB and pre-Caco2 prediction models were used to screen the potential active compounds from 551 constituents obtained above (the threshold setting: OB ≥ 30%, Caco-2 ≥ −0.40). There were a total of 283 compounds had good OB and Caco-2 values, including 28 components in astragali radix, 165 components in cinnamomi ramulus, 18 components in sparganii rhizoma, 43 components in curcumae rhizoma, 12 components in bolbostemmatis rhizoma, and 17 components in strychni semen. After deduplication, 223 compounds can be used as the potential active ingredients for QYSXC (the results are shown in Table S1 for 223 compounds). These compounds have been proved to have good pharmacological activity in anti-inflammatory and analgesic effects. For example, researches have showed that quercetin can effectively prevent the production of IL-1*β* and TNF-*α* in synovia fluid and can inhibit the occurrence of joint inflammation [26]; linolenic acid, taxifolin, and epigallocatechin have been reported to have anti-inflammatory and analgesic effects [27–29].

### 3.3. Target Recognition and Disease Association

According to the target recognition methods introduced in 2.3, 283 compounds and corresponding 473 targets were obtained (after deduplication). Then, all these targets were mapped to the diseases in TTD, CTD, and PharmGKB databases to further screen active ingredients and targets. The results showed that 137 constituents and 69 targets (after deduplication) were obtained in treatment with ONFH for QYSXC.

### 3.4. PPI Network Construction and Core Targets Determination

**Figure 1:**
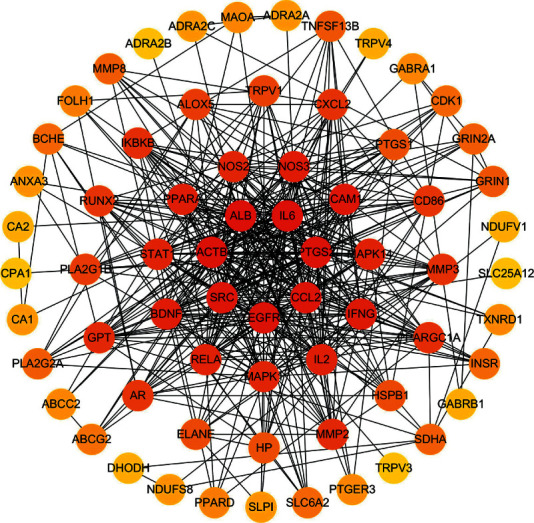
The protein-protein interaction (PPI) network for QYSXC in treatment with ONFH. Different colors represent varying degrees, and the darker the color, the greater the degree.

The PPI was obtained by STRING 11.0 platform, and the results were transfer to Cytoscape 3.8.0 software for redrawing. In the network, there were 2 of all nodes that have no connection with other nodes and have been removed. The results showed that these 67 targets can be regarded as the main targets for QYSXC, which had good connectivity in the PPI network. As shown in Figure 1, there were 67 nodes and 438 edges. Different colors represented varying degrees. The darker the color, the greater the degree. In other words, the degree value from the inside to the outside in the PPI network gradually decreases. These targets may be very important in the treatment of ONFH for QYSXC, especially the targets with large degree value, such as IL6, PTGS2, and ALB.

### 3.5. The Results of KEGG Pathway Enrichment Analysis

**Figure 2:**
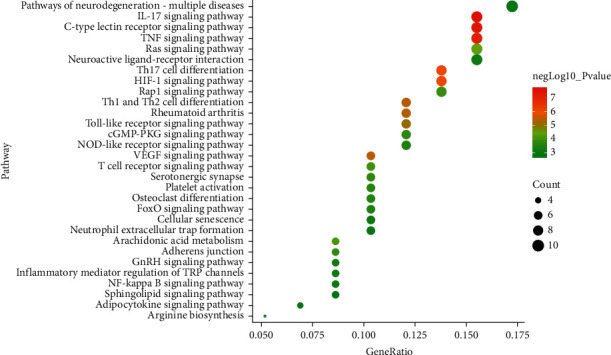
The results of KEGG pathway enrichment analysis by program R.

The 67 targets were significantly enriched in a total of 136 pathways with *P* < 0.05. The obviously unrelated pathways were removed, such as “Salmonella infection,” “Influenza A,” “Leishmaniasis,” and “Pathways in cancer.” Besides, these pathways in the top 30 according to the *P* value were visualized in Figure 2 by using program R. Some studies have showed that most of these pathways were closely related to ONFH, such as TNF signaling pathway, HIF-1 signaling pathway, PI3K-AKT signaling pathway, Toll-like receptor signaling pathway, and VEGF signaling pathway. In these pathways, TNF signaling pathway was one of the most important pathways with 9 enriched targets, including ICAM1, MAPK1, IL6, CCL2, PTGS2, MAPK14, RELA, IKBKB, and MMP3, and the *P* value is 8.06*E* − 08. The numbers of enriched targets in HIF-1 signaling pathway and PI3K-Akt signaling pathway are both 8. EGFR, MAPK1, IL6, RELA, IFNG, NOS3, NOS2, and INSR were enriched, respectively, in HIF-1 signaling pathway, and EGFR, MAPK1, IL6, RELA, NOS3, IKBKB, INSR, and IL2 were enriched in PI3K-Akt signaling pathway.

### 3.6. Network Construction and Analysis

**Figure 3:**
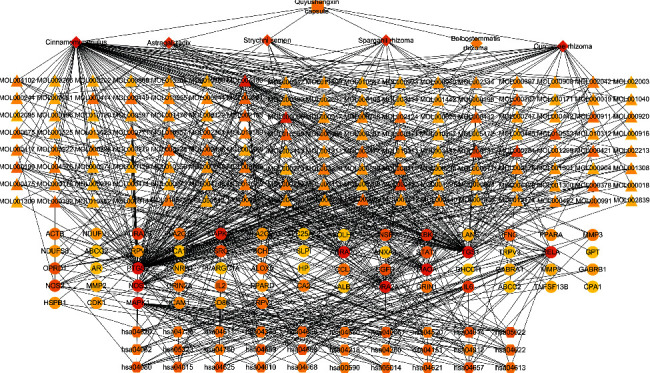
The herbs-compounds-targets-pathways (H-C-T-P) network for QYSXC in treatment with ONFH. Different colors represented varying degrees, and the darker the color, the greater the degree.

The herbs-compounds-targets-pathways (H-C-T-P) network was constructed for QYSXC in treatment with ONFH by Cytoscape 3.8.0 (Figure 3). The network was comprised of 241 nodes (6 herbs, 136 compounds, 67 targets, and 30 pathways) and 772 edges. According to the figure, the diamond represented the herbs, the triangle represented the compounds, the ellipse denoted the targets, and the hexagon represented the pathways. Different colors represented varying degrees, and the darker the color, the greater the degree. The topological properties have been analyzed by means of Network Analyzer plugin in the Cytoscape 3.8.0 software, including degree centrality (DC), betweenness centrality (BC), and closeness centrality (CC). These topological properties were important parameters of network analysis. They were able to reflect the importance of each node in the network. To be subjective, high topological properties mean more important compound or target in the network. As a result, 15 potential key compounds, 8 potential key targets, and 8 potential key signaling pathways were obtained for QYSXC in treatment with ONFH. The DC, BC, and CC properties of all these nodes are higher than the average (refer to Table 1).

The results of network analysis showed that quercetin had the best network topological properties, kaempferol next, and the third was linoleic acid. Quercetin and kaempferol were the main components of astragali radix. Quercetin has been used as an oral supplement for osteoarthritis (OA) because of its good anti-inflammatory and antioxidative effects, and it can alleviate symptoms and delay the progress of knee osteoarthritis [30]. In addition, the protective effects of quercetin were also observed in OA rat model, in which the degeneration of knee cartilage disappeared and the apoptosis of chondrocytes decreased [31]. Kaempferol can prevent or delay the progression of OA by reducing IL-1*β* and other proinflammatory ones in rat OA chondrocytes [32]. Wong et al. have reported that kaempferol can regulate ESR, NF-kB, and MAPK to inhibit adipogenesis, inflammation, oxidative stress, osteoclast autophagy, and osteoblast apoptosis and activate osteoblast autophagy to protect bone [33]. Linoleic acid has also been proved to play an anti-inflammatory role by regulating COX-2 (PTGS2) and had a good effect on osteoporosis, osteoarthritis, and inflammatory joint diseases [34].

In the potential key targets, PTGS2 and PTGS1 exhibited the particularly high DC, BC, and CC values. We found that all 15 key compounds were related to PTGS2 and PTGS1. Messenger RNA microarray analysis was carried out to analyze the differential expressed genes (DEGs) in OA synovial tissues and normal counterparts, and the results showed that PTGS1 was overexpressed in OA synovial cells and tissues compared with normal synovial cells [35]. There are many nonsteroidal anti-inflammatory drugs in the treatment of osteopathic medicine related to ONFH, which can specifically act on PTGS1 and PTGS2, such as naproxen, rofecoxib, and celecoxib [36–39]. NOS2 and NOS3 have also exhibited higher topology properties. NOS can produce a large amount of NO under pathological conditions. NO has been implicated as an important inflammatory mediator in arthritis. NO, as an inflammatory mediator and cytotoxic factor, can produce inflammation of the synovium, induce and directly cause the apoptosis of the synovium and chondrocytes, lead to the degradation of the cartilage matrix, and eventually result in the ONFH [40]. Moreover, other targets, such as RELA, IL6, and EGFR, can also participate in cell proliferation, transformation, and apoptosis and were closely related to the inflammation and immune response of important human pathophysiological processes.

According to the results of KEGG pathway enrichment analysis and network analysis, the pathways of neurodegeneration-multiple diseases and TNF signaling pathway may be the main pathways for QYSXC in treating ONFH. There are 10 targets were enriched in the pathways of neurodegeneration-multiple diseases, including PTGS2, NOS2, MAPK14, RELA, MAPK1, IL6, GRIN1, GRIN2A, NDUFS8, and NDUFV1. Studies have shown that neurodegeneration is related to some bone diseases, such as degenerative osteoarthritis and osteoporosis [41, 42]. In particular, a clinical study on mucopolysaccharidosis type III (MPS III) showed that the prevalence of ONFH in MPS III patients is extremely high. The main clinical feature of MPS III patients is progressive neurodegeneration [43]. These results suggested that the pathways of neurodegeneration-multiple diseases may be related to ONFH. There are 9 targets were enriched in the TNF signaling pathway, such as PTGS2, MAPK14, RELA, IKBKB, MMP3, MAPK1, IL6, ICAM1, and TNF-*α*. The occurrence of ONFH was closely related to autoimmune response. Clinical studies have found that the levels of many inflammatory related factors were increased in chondrocytes or serum of ONFH patients, such as IL-1*β*, TNF-*α*, PTGS2, and IL6 [44, 45]. In a clinical study, it was also found that the level of MMP3 in 57 ONHF patients was significantly higher than that in other bone diseases, including rapidly destructive arthrosis (RDA) and developmental dysplasia of the hip (DDH) [46].

### 3.7. Docking Results

**Figure 4:**
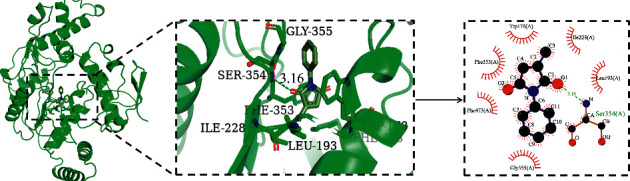
The molecular docking results between NOS3 and 2-methyl-N-phenylmaleimide.

To further explore the interaction between the key compounds and key targets, molecular docking was performed in this present study. The molecular docking was carried out between 4 key compounds (quercetin, kaempferol, linoleic acid, and 2-methyl-N-phenylmaleimide) with the highest degree centrality for QYSXC and 3 core targets, namely, PTGS2 (PDB ID: 5IKR), PTGS1 (PDB ID: 6Y3C), and NOS3 (PDB ID: 1M9R), respectively. The empirical threshold (-5.0 kcal/mol) mentioned was taken as the evaluation standard for evaluating the binding ability of key components and targets. If the docking binding energy is lower than the threshold, it showed that the binding ability between the target and the compound was stronger. The results showed that the binding abilities of most key ingredients and 3 key targets were higher than the empirical threshold, except PTGS1 with quercetin and PTGS1 with eugenol (see Table 2). Meanwhile, the interaction between the compounds and the targets was further analyzed in this study. Take the binding between NOS3 and 2-methyl-N-phenylmaleimide as an example (Figure 4); the ligand can bind in the active pocket composed of residues TRP178, PHE353, PHE473, GLY355, ILE228, and LEU193. There is a hydrogen bond between SER354 and 2-methyl-N-phenylmaleimide, and the binding energy is -7.04 kcal/mol.

All the results of systems pharmacology and molecular docking showed that the QYSXC can treat ONFH through multiple components, multiple targets, and multiple pathways. Subsequently, some targets were selected for further experimental verification for TNF signaling pathway, which was reported by literatures.

### 3.8. QYSXC Treatment Increases Serum Levels of Key Proinflammatory Cytokines

**Figure 5:**
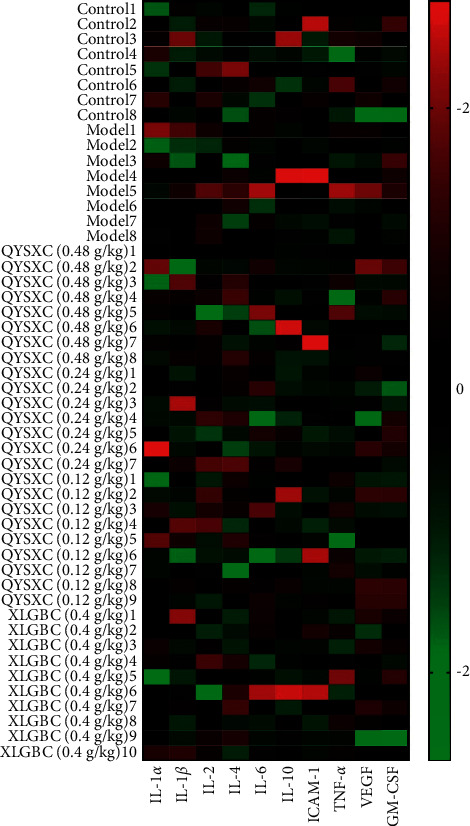
The heatmap of 10 protein indexes in 50 samples by Me V software (control group: *n* = 8; model group: *n* = 8; 0.48 g/kg QYSXC group: *n* = 8; 0.24 g/kg QYSXC group: *n* = 7; 0.12 g/kg QYSXC group: *n* = 9; XLGBC group: *n* = 10).

The heatmap of 10 protein indexes in 50 samples was drawn by Me V software (see Figure 5). In this figure, diverse colors represented the level of concentration, the red represented high concentration, the green represented low concentration, and the white represented the middle level. The stronger the red or green, the higher or lower the protein concentration in the samples. The heatmap intuitively illustrated the contrast regulating expression of the chip detection proteins in different groups. According to the heatmap, IL-1*α*, IL-1*β*, IL-2, IL-4, IL-6, IL-10, ICAM-1, and TNF-*α* were selected for further statistical analysis.

**Figure 6:**
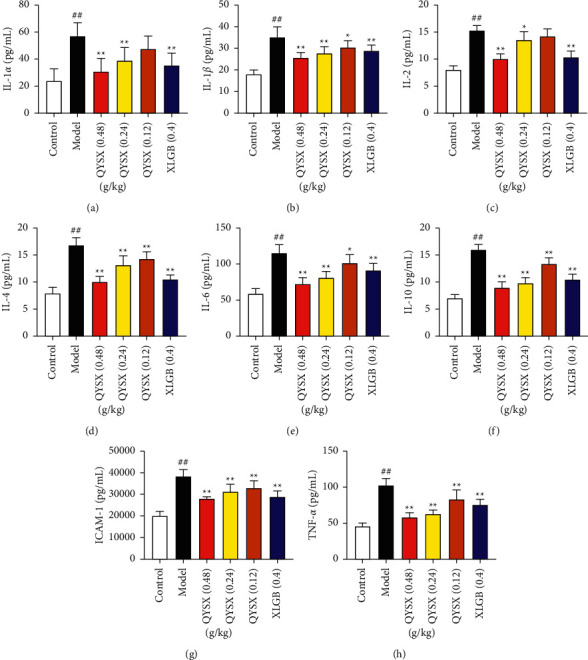
QYSXC treatment decreased serum levels of key proinflammatory cytokines. All data were expressed as the mean ± standard deviation (SD) (blank control group: *n* = 8; model group: *n* = 8; 0.48 g/kg QYSXC group: *n* = 8; 0.24 g/kg QYSXC group: *n* = 7; 0.12 g/kg QYSXC group: *n* = 9; XLGBC group: *n* = 10). ^*∗*^ indicates *P* < 0.05, ^*∗∗*^ indicates *P* < 0.01., for the QYSXC groups vs the model group; ^##^ indicates *P* < 0.01, for the model group vs. the blank control group.

Figure 6 showed the statistical analysis results of IL-1*α*, IL-1*β*, IL-2, IL-4, IL-6, IL-10, ICAM-1, and TNF-*α*. As shown in Figure 6, compared with the model group, the serum levels of all cytokines were significantly downregulated in most of the QYSXC groups, except IL-1*α* (0.12 g/kg QYSX group, Figure 6(a)) and IL-2 (0.12 g/kg QYSX group, Figure 6(c)). Take IL-6 as example (see Figure 6(e)); regarding the average levels of concentration, the blank control group was 59.2 ± 6.8 pg/mL, the model group was 114.7 ± 12.8 pg/mL, QYSXC (0.48 g/kg) group was 72.6 ± 8.9 pg/mL, QYSXC (0.24 g/kg) group was 81.5 ± 8.3 pg/mL, and QYSXC (0.12 g/kg) group was 101.5 ± 11.6 pg/mL. Compared with the model group, the levels of IL-6 in all QYSXC groups were significantly decreased, *P* < 0.01 in the high and medium dose groups, and *P* < 0.05 in the low dose group, respectively. The results showed that QYSXC could inhibit the serum levels of IL-1*α*, IL-1*β*, IL-2, IL-4, IL-6, IL-10, ICAM-1, and TNF-*α*, and QYSX capsule may prevent and cure ONFH by inhibiting the expression of inflammatory factors. ONFH was a main cause of hip diseases in young adults and is frequently observed in patients treated with high dose of glucocorticoids. There have been various hypotheses about the pathogenesis of ONFH [47]. Studies have shown that inflammation and inflammatory cytokines played a crucial role in many diseases and were also a crucial contributors in the ONFH [44]. A large number of inflammatory factors were released, which drove the immune cells to induce infiltration of the great vascular region. They also induced the immune inflammatory response in the vascular region, continuously damaged the blood vessels, and finally led to necrosis of the femoral head [48]. This work may provide a new theoretical basis for the clinical treatment of ONFH.

## 4. Conclusion

In the present study, an integrated approach was used to investigate the mechanism of QYSXC in the treatment of ONFH, which contained systems pharmacology, molecular docking, and chip experiment. The results showed that 15 potential key compounds, 8 potential key targets, and 8 potential key signaling pathways were determined for QYSXC in treatment with ONFH. And then, the molecular docking and chip experiment have been carried out to verify some key targets. In short, QYSXC can effectively treat ONFH by means of multiple components, multiple targets, and multiple pathways. The results of this work may provide a theoretical basis for further research on the molecular mechanism of QYSXC in the treatment of ONFH.

## Figures and Tables

**Table 1:** The network topological properties of the potential key compounds, targets, and pathways for QYSXC in treatment with ONFH.

No.	Node	Node name	Degree centrality (DC)	Betweenness centrality (BC)	Closeness centrality (CC)
1	MOL000098	Quercetin	25	0.1284	0.4691
2	MOL000422	Kaempferol	22	0.0752	0.4296
3	MOL000131	Linoleic acid	12	0.0268	0.4130
4	MOL010552	2-Methyl-N-phenylmaleimide	10	0.0195	0.4073
5	MOL000378	7-O-Methylisomucronulatol	9	0.0113	0.4087
6	MOL000421	Nicotinic acid	7	0.0119	0.4116
7	MOL000432	Linolenic acid	7	0.0191	0.3846
8	MOL000449	Stigmasterol	7	0.0188	0.4073
9	MOL000771	P-Coumaric acid	6	0.0100	0.4059
10	MOL000417	Calycosin	6	0.0055	0.4017
11	MOL000675	Oleic acid	6	0.0059	0.4234
12	MOL000991	Cinnamaldehyde	5	0.0043	0.3990
13	MOL002042	Thymol	5	0.0055	0.4003
14	MOL003319	4-Carboxymethylphenol	5	0.0124	0.4045
15	MOL004576	Taxifolin	5	0.0078	0.4059
16	PTGS2	Prostaglandin G/H synthase 2	120	0.4175	0.5802
17	PTGS1	Prostaglandin G/H synthase 1	78	0.1538	0.4825
18	NOS3	Nitric oxide synthase, endothelial	31	0.0241	0.3577
19	ADRA2C	Alpha-2C adrenergic receptor	30	0.0219	0.3461
20	MAOA	Amine oxidase (flavin-containing) A	27	0.0265	0.3666
21	RELA	Transcription factor p65	26	0.0329	0.3666
22	MAPK1	Mitogen-activated protein kinase 1	25	0.0474	0.3784
23	MAPK14	Mitogen-activated protein kinase 14	24	0.0368	0.3451
24	hsa05022	Pathways of neurodegeneration-multiple diseases	10	0.0173	0.4116
25	hsa04668	TNF signaling pathway	9	0.0138	0.4087
26	hsa04625	C-type lectin receptor signaling pathway	8	0.0138	0.4087
27	hsa04657	IL-17 signaling pathway	8	0.0132	0.4087
28	hsa04022	cGMP-PKG signaling pathway	7	0.0101	0.3250
29	hsa04726	Serotonergic synapse	6	0.0088	0.4174
30	hsa04370	VEGF signaling pathway	6	0.0092	0.4017
31	hsa04611	Platelet activation	6	0.0065	0.3621

**Table 2:** The docking energy results of the complex between some key compounds and targets for QYSXC in treatment with ONFH.

Protein name	Gene name	PDB ID	Ligand name	Binding energy (kcal/mol)
Prostaglandin G/H synthase 2	PTGS2	5IKR	Quercetin	−5.28
			Kaempferol	−6.03
			Linoleic acid	−6.55
			2-Methyl-N-phenylmaleimide	−6.65

Prostaglandin G/H synthase 1	PTGS1	6Y3C	Quercetin	−4.95
			Kaempferol	−5.53
			Linoleic acid	−6.15
			2-Methyl-N-phenylmaleimide	−6.51

Nitric oxide synthase, endothelial	NOS3	1M9R	Quercetin	−5.88
			Kaempferol	−6.46
			Linoleic acid	−6.45
			2-Methyl-N-phenylmaleimide	−7.04

## Data Availability

The data used to support the findings of this study are included in Supplementary Materials.

## Supplementary Materials

Supplementary Materials The detailed information of chemical compounds of herbs is presented in Table S1.
