# Supplementary material for: The System Research of the Molecular Mechanism of Quyushengxin Capsule in the Treatment of Osteonecrosis of the Femoral Head
Source: Evid Based Complement Alternat Med. 2022 Jan 11;2022:2968075. doi: 10.1155/2022/2968075 (PMC8767393; doi:10.1155/2022/2968075)
Supplement: Supplementary Materials — The detailed information of chemical compounds of herbs is presented in Table S1. [file 2968075.f1.docx]

**The system research of the molecular mechanism of Quyushengxin capsule in the treatment of osteonecrosis of the femeral head**

Xia Du^1, 2#^, Lintao Zhao^1#^, Yuan Qiao^1^, Yuan Liu^1^, Dong Guo^1,⁎^

^1^Institute of Traditional Chinese Medicine, Shaanxi Academy of Traditional Chinese Medicine, Xi'an, Shaanxi, 710003, China

^2^Center for Post-doctoral Studies, China Academy of Chinese Medical Sciences, Beijing, 100700, China

^#^These authors have contributed equally to this work.

^*^Corresponding author.

Email: 787422146@qq.com

**Table S1. The ADME properties of 223 potential active ingredients for QYSXC**

| No. | Mol ID | Compound | OB (%) | Caco-2 | Herb |
| --- | --- | --- | --- | --- | --- |
| 1 | MOL000114 | Vanillic acid | 35.47 | 0.43 | Astragali radix |
| 2 | MOL000131 | Linoleic Acid | 41.9 | 1.16 | Astragali radix, Cinnamomi ramulus |
| 3 | MOL000211 | Mairin | 55.38 | 0.73 | Astragali radix |
| 4 | MOL000239 | Jaranol | 50.83 | 0.61 | Astragali radix |
| 5 | MOL000296 | Hederagenin | 36.91 | 1.32 | Astragali radix, Sparganii rhizoma, Curcumae rhizoma |
| 6 | MOL000033 | (3S,8S,9S,10R,13R,14S,17R)-10,13-dimethyl-17-[(2R,5S)-5-propan-2-yloctan-2-yl]-2,3,4,7,8,9,11,12,14,15,16,17-dodecahydro-1H-cyclopenta[a]phenanthren-3-ol | 36.23 | 1.45 | Astragali radix |
| 7 | MOL000354 | Isorhamnetin | 49.6 | 0.31 | Astragali radix |
| 8 | MOL000371 | 3,9-di-O-methylnissolin | 53.74 | 1.18 | Astragali radix |
| 9 | MOL000372 | 3-Hydroxy-2-picoline | 62.47 | 1.05 | Astragali radix |
| 10 | MOL000378 | 7-O-methylisomucronulatol | 74.69 | 1.08 | Astragali radix |
| 11 | MOL000380 | (6aR,11aR)-9,10-dimethoxy-6a,11a-dihydro-6H-benzofurano[3,2-c]chromen-3-ol | 64.26 | 0.93 | Astragali radix |
| 12 | MOL000381 | 13-hydroxy-9,11-octadecadienoic acid | 35.6 | 0.44 | Astragali radix |

**Continued Table S1. The ADME properties of 223 potential active ingredients for QYSXC**

| 13 | MOL005928 | Isoferulic acid | 50.83 | 0.49 | Astragali radix |
| --- | --- | --- | --- | --- | --- |
| 14 | MOL000387 | Bifendate | 31.1 | 0.15 | Astragali radix |
| 15 | MOL000389 | FERULIC ACID (CIS) | 54.97 | 0.53 | Astragali radix |
| 16 | MOL000392 | Formononetin | 69.67 | 0.78 | Astragali radix, Sparganii rhizoma |
| 17 | MOL000397 | Cis-p-Coumarate | 45.98 | 0.46 | Astragali radix |
| 18 | MOL000398 | Isoflavanone | 109.99 | 0.53 | Astragali radix |
| 19 | MOL000414 | Caffeate | 54.97 | 0.27 | Astragali radix |
| 20 | MOL000417 | Calycosin | 47.75 | 0.52 | Astragali radix |
| 21 | MOL000421 | Nicotinic acid | 47.65 | 0.34 | Astragali radix |
| 22 | MOL000422 | Kaempferol | 41.88 | 0.26 | Astragali radix |
| 23 | MOL000432 | Linolenic acid | 45.01 | 1.21 | Astragali radix |
| 24 | MOL000436 | (Z)-1-(2,4-dihydroxyphenyl)-3-(4-hydroxyphenyl)prop-2-en-1-one | 87.51 | 0.2 | Astragali radix |
| 25 | MOL000438 | (3R)-3-(2-hydroxy-3,4-dimethoxyphenyl)chroman-7-ol | 67.67 | 0.96 | Astragali radix |
| 26 | MOL000442 | 1,7-Dihydroxy-3,9-dimethoxy pterocarpene | 39.05 | 0.89 | Astragali radix |
| 27 | MOL000061 | Prolinum | 77.57 | 0.22 | Astragali radix |

**Continued Table S1. The ADME properties of 223 potential active ingredients for QYSXC**

| 28 | MOL000098 | Quercetin | 46.43 | 0.05 | Astragali radix |
| --- | --- | --- | --- | --- | --- |
| 29 | MOL000103 | PHB | 30.15 | 0.39 | Cinnamomi ramulus, Strychni semen |
| 30 | MOL010545 | 2-Methoxycinnamic Acid | 31.79 | 0.7 | Cinnamomi ramulus |
| 31 | MOL010547 | 2-Phenylpropenal | 30.68 | 1.38 | Cinnamomi ramulus |
| 32 | MOL010549 | m-Formylphenol | 30.97 | 0.83 | Cinnamomi ramulus |
| 33 | MOL010550 | 4-Methyl-2-(1,5-dimethyl-4-hexenyl)-3-cyclohexen-1-ol | 39.63 | 1.44 | Cinnamomi ramulus |
| 34 | MOL010551 | DMEP | 55.66 | 0.15 | Cinnamomi ramulus |
| 35 | MOL010552 | 2-Methyl-N-phenylmaleimide | 87.36 | 0.77 | Cinnamomi ramulus |
| 36 | MOL010553 | β-Bisabolene | 33.46 | 1.93 | Cinnamomi ramulus |
| 37 | MOL010554 | 694-87-1 | 48.07 | 1.86 | Cinnamomi ramulus |
| 38 | MOL010555 | 1,2-Dibenzoylethane | 57.97 | 0.98 | Cinnamomi ramulus |
| 39 | MOL010556 | WLN: RVO2R | 31.04 | 1.35 | Cinnamomi ramulus |
| 40 | MOL010558 | Substance H 36 | 41.58 | 0.8 | Cinnamomi ramulus |
| 41 | MOL010559 | 2-Coumarinate | 60.17 | 0.46 | Cinnamomi ramulus |
| 42 | MOL010560 | WLN: VHO2R | 30 | 1.23 | Cinnamomi ramulus |
| 43 | MOL010561 | 2-Methoxyphenylacetone | 39.61 | 1.22 | Cinnamomi ramulus |

**Continued Table S1. The ADME properties of 223 potential active ingredients for QYSXC**

| 44 | MOL010562 | Hydro Cinnamicacid | 36.71 | 0.88 | Cinnamomi ramulus |
| --- | --- | --- | --- | --- | --- |
| 45 | MOL010563 | Methyl (Z)-cinnamate | 37.2 | 1.31 | Cinnamomi ramulus |
| 46 | MOL010564 | NK | 32.1 | 0.89 | Cinnamomi ramulus |
| 47 | MOL010957 | (Z)-Ethyl cinnamate | 37.28 | 1.27 | Cinnamomi ramulus |
| 48 | MOL001121 | 19894-97-4 | 49.98 | 1.25 | Cinnamomi ramulus |
| 49 | MOL000118 | (L)-alpha-Terpineol | 48.8 | 1.39 | Cinnamomi ramulus, Curcumae rhizoma |
| 50 | MOL001217 | ()-Bornyl acetate | 65.55 | 1.3 | Cinnamomi ramulus |
| 51 | MOL000125 | (-)-alpha-Pinene | 46.25 | 1.85 | Cinnamomi ramulus |
| 52 | MOL000126 | (-)-nopinene | 44.84 | 1.8 | Cinnamomi ramulus |
| 53 | MOL000130 | CAM | 67.17 | 1.29 | Cinnamomi ramulus, Curcumae rhizoma |
| 54 | MOL001300 | PEL | 44.03 | 1.11 | Cinnamomi ramulus, Sparganii rhizoma |
| 55 | MOL001301 | Cis-Zimtsaeure | 38.19 | 0.91 | Cinnamomi ramulus, Sparganii rhizoma |
| 56 | MOL001452 | Protocatechualdehyde | 38.35 | 0.43 | Cinnamomi ramulus |
| 57 | MOL001578 | Hypnon | 48.19 | 1.36 | Cinnamomi ramulus |
| 58 | MOL001599 | α-cubebol | 64.81 | 1.32 | Cinnamomi ramulus |
| 59 | MOL001736 | (-)-Taxifolin | 60.51 | -0.24 | Cinnamomi ramulus |

**Continued Table S1. The ADME properties of 223 potential active ingredients for QYSXC**

| 60 | MOL001746 | ELD | 31.2 | 1.26 | Cinnamomi ramulus |
| --- | --- | --- | --- | --- | --- |
| 61 | MOL000019 | D-Camphene | 34.98 | 1.81 | Cinnamomi ramulus, Curcumae rhizoma |
| 62 | MOL002024 | (1S,4R)-1,7,7-trimethylbicyclo[2.2.1]hept-2-ene | 39.62 | 1.8 | Cinnamomi ramulus |
| 63 | MOL002153 | 1H-Cycloprop(e)azulen-7-ol, decahydro-1,1,7-trimethyl-4-methylene-, (1aR-(1aalpha,4aalpha,7beta,7abeta,7balpha))- | 82.33 | 1.37 | Cinnamomi ramulus |
| 64 | MOL002163 | WLN: 2VR | 60.17 | 1.45 | Cinnamomi ramulus |
| 65 | MOL000219 | BOX | 31.55 | 0.54 | Cinnamomi ramulus |
| 66 | MOL002193 | Dihydroeugenol | 62.43 | 1.42 | Cinnamomi ramulus |
| 67 | MOL002213 | (Z)-1,3-di(phenyl)prop-2-en-1-one | 47.27 | 1.48 | Cinnamomi ramulus |
| 68 | MOL002225 | Styrone | 38.35 | 1.14 | Cinnamomi ramulus |
| 69 | MOL000234 | L-Limonen | 38.09 | 1.83 | Cinnamomi ramulus |
| 70 | MOL002361 | Terragon | 36.59 | 1.72 | Cinnamomi ramulus |
| 71 | MOL000244 | ()-Borneol | 81.8 | 1.22 | Cinnamomi ramulus, Curcumae rhizoma |
| 72 | MOL000247 | (Z,Z)-farnesol | 41.14 | 1.25 | Cinnamomi ramulus |
| 73 | MOL000254 | Eugenol | 56.24 | 1.35 | Cinnamomi ramulus |

**Continued Table S1. The ADME properties of 223 potential active ingredients for QYSXC**

| 74 | MOL002888 | o-Anisaldehyde | 57.94 | 1.06 | Cinnamomi ramulus |
| --- | --- | --- | --- | --- | --- |
| 75 | MOL003047 | [(1S)-endo]-(-)-Borneol | 83.54 | 1.22 | Cinnamomi ramulus |
| 76 | MOL000340 | HCI | 35.83 | 0.92 | Cinnamomi ramulus |
| 77 | MOL003525 | Pyruvophenone | 35.93 | 0.92 | Cinnamomi ramulus |
| 78 | MOL003537 | T-Muurolol | 30.41 | 1.36 | Cinnamomi ramulus |
| 79 | MOL003541 | ()-alpha-Longipinene | 57.47 | 1.81 | Cinnamomi ramulus |
| 80 | MOL000358 | Beta-sitosterol | 36.91 | 1.32 | Cinnamomi ramulus, Sparganii rhizoma, Bolbostemmatis rhizoma |
| 81 | MOL000359 | Sitosterol | 36.91 | 1.32 | Cinnamomi ramulus, Bolbostemmatis rhizoma |
| 82 | MOL000475 | Anethole | 32.49 | 1.75 | Cinnamomi ramulus |
| 83 | MOL000492 | (+)-Catechin | 54.83 | -0.03 | Cinnamomi ramulus, Strychni semen |
| 84 | MOL002003 | (-)-Caryophyllene oxide | 32.67 | 1.58 | Cinnamomi ramulus |
| 85 | MOL000608 | ()-Terpinen-4-ol | 81.41 | 1.36 | Cinnamomi ramulus |
| 86 | MOL000612 | (-)-Alpha-cedrene | 55.56 | 1.81 | Cinnamomi ramulus |
| 87 | MOL000666 | Hexanal | 55.71 | 1.25 | Cinnamomi ramulus |
| 88 | MOL000671 | ()-Menthol | 59.33 | 1.27 | Cinnamomi ramulus |

**Continued Table S1. The ADME properties of 223 potential active ingredients for QYSXC**

| 89 | MOL000676 | DBP | 64.54 | 0.8 | Cinnamomi ramulus |
| --- | --- | --- | --- | --- | --- |
| 90 | MOL000708 | WLN: VHR | 32.63 | 1.32 | Cinnamomi ramulus |
| 91 | MOL000714 | Hyacinthin | 38.65 | 1.31 | Cinnamomi ramulus |
| 92 | MOL000073 | Ent-Epicatechin | 48.96 | 0.02 | Cinnamomi ramulus |
| 93 | MOL000771 | P-coumaric acid | 43.29 | 0.46 | Cinnamomi ramulus |
| 94 | MOL008656 | (1R,3R,4S)-3,4-dimethylcyclohexan-1-ol | 43.57 | 1.26 | Cinnamomi ramulus |
| 95 | MOL008668 | 2-ethoxypropanol | 47.47 | 0.67 | Cinnamomi ramulus |
| 96 | MOL000991 | Cinnamaldehyde | 31.99 | 1.35 | Cinnamomi ramulus |
| 97 | MOL000018 | (+/-)-Isoborneol | 86.98 | 1.27 | Cinnamomi ramulus, Curcumae rhizoma |
| 98 | MOL000023 | Hemo-sol | 39.84 | 1.83 | Cinnamomi ramulus, Curcumae rhizoma |
| 99 | MOL000025 | α-Longipinene | 53.26 | 1.83 | Cinnamomi ramulus |
| 100 | MOL000057 | DIBP | 49.63 | 0.85 | Cinnamomi ramulus |
| 101 | MOL000122 | 1,8-cineole | 39.73 | 1.57 | Cinnamomi ramulus |
| 102 | MOL000171 | Guaiol | 38.77 | 1.36 | Cinnamomi ramulus |
| 103 | MOL000196 | L-Bornyl acetate | 65.52 | 1.29 | Cinnamomi ramulus |
| 104 | MOL000198 | (R)-linalool | 39.8 | 1.33 | Cinnamomi ramulus |

**Continued Table S1. The ADME properties of 223 potential active ingredients for QYSXC**

| 105 | MOL000199 | Safrol | 45.34 | 1.44 | Cinnamomi ramulus |
| --- | --- | --- | --- | --- | --- |
| 106 | MOL000202 | Moslene | 33.02 | 1.88 | Cinnamomi ramulus |
| 107 | MOL000207 | Methyleugenol | 73.36 | 1.47 | Cinnamomi ramulus |
| 108 | MOL000208 | ()-Aromadendrene | 55.74 | 1.81 | Cinnamomi ramulus, Curcumae rhizoma |
| 109 | MOL000257 | (-)-Beta-Phellandrene | 40.44 | 1.83 | Cinnamomi ramulus |
| 110 | MOL000259 | O-Thymol | 43.28 | 1.58 | Cinnamomi ramulus |
| 111 | MOL000266 | Beta-Cubebene | 32.81 | 1.83 | Cinnamomi ramulus |
| 112 | MOL000270 | CHEBI:7 | 45.2 | 1.84 | Cinnamomi ramulus |
| 113 | MOL000474 | (-)-Epoxycaryophyllene | 35.94 | 1.57 | Cinnamomi ramulus, Curcumae rhizoma |
| 114 | MOL000597 | Neryl acetate | 57.47 | 1.25 | Cinnamomi ramulus |
| 115 | MOL000675 | Oleic acid | 33.13 | 1.17 | Cinnamomi ramulus |
| 116 | MOL000699 | M-Cymol | 48.85 | 1.88 | Cinnamomi ramulus |
| 117 | MOL000905 | ()-Beta-Pinene | 44.77 | 1.85 | Cinnamomi ramulus, Curcumae rhizoma |
| 118 | MOL000911 | Terpilene | 33.95 | 1.84 | Cinnamomi ramulus, Curcumae rhizoma |
| 119 | MOL000914 | (5S)-1-isopropyl-4-methylbicyclo[3.1.0]hex-3-ene | 47.13 | 1.81 | Cinnamomi ramulus, Curcumae rhizoma |
| 120 | MOL000922 | (R)-p-Menth-1-en-4-ol | 32.16 | 1.33 | Cinnamomi ramulus, Curcumae rhizoma |

**Continued Table S1. The ADME properties of 223 potential active ingredients for QYSXC**

| 121 | MOL000935 | Hepanal | 53.83 | 1.86 | Cinnamomi ramulus, Curcumae rhizoma |
| --- | --- | --- | --- | --- | --- |
| 122 | MOL000937 | 58870_FLUKA | 49.01 | 1.82 | Cinnamomi ramulus, Curcumae rhizoma |
| 123 | MOL000942 | (1R,4S,4aR,8aR)-4-isopropyl-1,6-dimethyl-3,4,4a,7,8,8a-hexahydro-2H-naphthalen-1-ol | 31.67 | 1.32 | Cinnamomi ramulus, Curcumae rhizoma |
| 124 | MOL000974 | Cuminal | 38.29 | 1.39 | Cinnamomi ramulus |
| 125 | MOL001179 | (8722;)-Alloaromadendrene | 54.04 | 1.81 | Cinnamomi ramulus |
| 126 | MOL001237 | O-Acetyltoluene | 38.96 | 1.47 | Cinnamomi ramulus |
| 127 | MOL001442 | Phytol | 33.82 | 1.23 | Cinnamomi ramulus |
| 128 | MOL002040 | (1S,4R)-fenchone | 72.64 | 1.35 | Cinnamomi ramulus |
| 129 | MOL002042 | Thymol | 41.47 | 1.6 | Cinnamomi ramulus |
| 130 | MOL002046 | Hexanoic acid | 73.08 | 0.8 | Cinnamomi ramulus |
| 131 | MOL002095 | DEP | 52.19 | 0.72 | Cinnamomi ramulus |
| 132 | MOL002124 | Beta-asarone | 35.61 | 1.45 | Cinnamomi ramulus |
| 133 | MOL002334 | Homocresol | 35.9 | 1.36 | Cinnamomi ramulus |
| 134 | MOL002453 | (-)-Comphene | 34.98 | 1.81 | Cinnamomi ramulus |
| 135 | MOL002691 | Iva | 62.17 | 0.82 | Cinnamomi ramulus |

**Continued Table S1. The ADME properties of 223 potential active ingredients for QYSXC**

| 136 | MOL002839 | Chavicol | 44.19 | 1.6 | Cinnamomi ramulus |
| --- | --- | --- | --- | --- | --- |
| 137 | MOL002983 | Guasol | 51.6 | 1.28 | Cinnamomi ramulus |
| 138 | MOL002998 | IPH | 36.05 | 1.5 | Cinnamomi ramulus |
| 139 | MOL003050 | Nonanoic acid | 40.51 | 0.92 | Cinnamomi ramulus |
| 140 | MOL003177 | Syringaldehyde | 67.06 | 0.71 | Cinnamomi ramulus |
| 141 | MOL003504 | Tolualdehydes | 44.18 | 1.39 | Cinnamomi ramulus |
| 142 | MOL003521 | Isohomogenol | 32.61 | 1.49 | Cinnamomi ramulus |
| 143 | MOL003524 | Benzenepropanol | 36.57 | 1.18 | Cinnamomi ramulus |
| 144 | MOL004102 | 2-Coumarate | 53.6 | 0.48 | Cinnamomi ramulus |
| 145 | MOL004479 | O-cresol | 62.45 | 1.57 | Cinnamomi ramulus |
| 146 | MOL004480 | Acetic acid | 47.87 | 0.42 | Cinnamomi ramulus |
| 147 | MOL004576 | Taxifolin | 57.84 | -0.23 | Cinnamomi ramulus |
| 148 | MOL004707 | Tau-cadinol | 36.51 | 1.31 | Cinnamomi ramulus |
| 149 | MOL005472 | 1,2-Benzenedicarboxylicacid, mono(2-ethyl) hexylester | 55.17 | 0.57 | Cinnamomi ramulus |
| 150 | MOL005979 | Papite | 31.04 | 1.07 | Cinnamomi ramulus |
| 151 | MOL006219 | Clorius | 45.99 | 1.25 | Cinnamomi ramulus |

**Continued Table S1. The ADME properties of 223 potential active ingredients for QYSXC**

| 152 | MOL007946 | 19435-97-3 | 33.04 | 1.32 | Cinnamomi ramulus |
| --- | --- | --- | --- | --- | --- |
| 153 | MOL010120 | (1S,4R,4aR,8aR)-1-isopropyl-4,7-dimethyl-2,3,4,5,6,8a-hexahydro-1H-naphthalen-4a-ol | 62.54 | 1.49 | Cinnamomi ramulus |
| 154 | MOL010257 | Isoamyl benzoate | 52.2 | 1.29 | Cinnamomi ramulus |
| 155 | MOL010380 | ()-alpha-Funebrene | 49.41 | 1.81 | Cinnamomi ramulus |
| 156 | MOL011169 | Peroxyergosterol | 44.39 | 0.86 | Cinnamomi ramulus |
| 157 | MOL011432 | (1R,3R,5R)-6,6-dimethyl-2-methylene-3-norpinanol | 51.38 | 1.27 | Cinnamomi ramulus |
| 158 | MOL011848 | D-Piperitone | 48.75 | 1.42 | Cinnamomi ramulus |
| 159 | MOL011899 | Eremophilene | 34.6 | 1.85 | Cinnamomi ramulus |
| 160 | MOL012174 | O-Anisic acid | 73.92 | 0.83 | Cinnamomi ramulus |
| 161 | MOL012344 | (4aR,9aS)-2,9,9-trimethyl-5-methylene-4,4a,6,7,8,9a-hexahydro-3H-benzo[7]annulene | 46.05 | 1.82 | Cinnamomi ramulus |
| 162 | MOL013023 | Biosol | 44.42 | 1.59 | Cinnamomi ramulus |
| 163 | MOL013205 | P-Methoxycinnamaldehyde | 59.64 | 1.12 | Cinnamomi ramulus |
| 164 | MOL001297 | Trans-gondoic acid | 30.70 | 1.19 | Sparganii rhizoma |
| 165 | MOL001298 | Dehydrocostus lactone | 58.57 | 1.27 | Sparganii rhizoma |

**Continued Table S1. The ADME properties of 223 potential active ingredients for QYSXC**

| 166 | MOL001299 | (1S,2S)-1,2-bis(2-furyl)ethane-1,2-diol | 32.52 | 0.32 | Sparganii rhizoma |
| --- | --- | --- | --- | --- | --- |
| 167 | MOL001304 | 2-ACETYLPYRROLE | 58.37 | 1.16 | Sparganii rhizoma, Bolbostemmatis rhizoma |
| 168 | MOL001305 | 3,4-dihydro-8-hydroxy-3-methyl-1H-2-benzopyran-4-one | 34.84 | 0.58 | Sparganii rhizoma |
| 169 | MOL001308 | Oleic acid | 33.13 | 1.14 | Sparganii rhizoma |
| 170 | MOL001309 | 6-Methylolpyridin-3-ol | 47.53 | 0.34 | Sparganii rhizoma |
| 171 | MOL001311 | Ricineic acid | 41.70 | 1.18 | Sparganii rhizoma |
| 172 | MOL001312 | 9-HEXADECENOIC ACID | 35.78 | 1.1 | Sparganii rhizoma |
| 173 | MOL000449 | Stigmasterol | 43.83 | 1.44 | Sparganii rhizoma, Strychni semen |
| 174 | MOL000748 | HMF | 45.07 | 0.05 | Sparganii rhizoma, Bolbostemmatis rhizoma |
| 175 | MOL000749 | Linoleic | 41.9 | 1.23 | Sparganii rhizoma |
| 176 | MOL000268 | (1S,5S)-1-isopropyl-4-methylenebicyclo[3.1.0]hexane | 46.21 | 1.83 | Curcumae rhizoma |
| 177 | MOL000048 | (5E,9Z)-3,6,10-trimethyl-4,7,8,11-tetrahydrocyclodeca[b]furan | 43.17 | 1.77 | Curcumae rhizoma |
| 178 | MOL000485 | TMH | 46.25 | 1.82 | Curcumae rhizoma |
| 179 | MOL000889 | Isocurcumenol | 97.67 | 1.11 | Curcumae rhizoma |
| 180 | MOL000891 | (1R,10R)-epoxy-1,10-dihydrocurdione | 36.73 | 0.68 | Curcumae rhizoma |

**Continued Table S1. The ADME properties of 223 potential active ingredients for QYSXC**

| 181 | MOL000894 | Turmerone | 32.98 | 1.33 | Curcumae rhizoma |
| --- | --- | --- | --- | --- | --- |
| 182 | MOL000897 | (3S,3aR,8aR)-3,8a-dihydroxy-5-isopropylidene-3,8-dimethyl-1,2,3a,4-tetrahydroazulen-6-one | 38.7 | 0.1 | Curcumae rhizoma |
| 183 | MOL000898 | (1S,6R,7R)-4-isopropylidene-1-methyl-7-(3-oxobutyl)norcaran-3-one | 34.17 | 0.91 | Curcumae rhizoma |
| 184 | MOL000899 | Furanodiene | 45.11 | 1.77 | Curcumae rhizoma |
| 185 | MOL000900 | (5R,6R)-5-isopropenyl-3,6-dimethyl-6-vinyl-5,7-dihydrobenzofuran-4-one | 57.05 | 1.28 | Curcumae rhizoma |
| 186 | MOL000901 | BRN 3094585 | 87.82 | 1.14 | Curcumae rhizoma |
| 187 | MOL000902 | Curcumol | 103.55 | 1.12 | Curcumae rhizoma |
| 188 | MOL000903 | Gweicurculactone | 42.92 | 1.37 | Curcumae rhizoma |
| 189 | MOL000904 | Cineole | 59.96 | 1.55 | Curcumae rhizoma |
| 190 | MOL000906 | Wenjine | 47.93 | 0.3 | Curcumae rhizoma |
| 191 | MOL000910 | Germacron | 32.5 | 1.33 | Curcumae rhizoma |
| 192 | MOL000913 | Tricyclene | 36.11 | 1.8 | Curcumae rhizoma |
| 193 | MOL000915 | (1S,10S),(4S,5S)-germacrone-1(10),4-diepoxide | 30.48 | 0.61 | Curcumae rhizoma |

**Continued Table S1. The ADME properties of 223 potential active ingredients for QYSXC**

| 194 | MOL000916 | Car-3-ene | 45.15 | 1.85 | Curcumae rhizoma |
| --- | --- | --- | --- | --- | --- |
| 195 | MOL000917 | Cineole | 59.96 | 1.55 | Curcumae rhizoma |
| 196 | MOL000920 | LINALOOL (D) | 38.29 | 1.29 | Curcumae rhizoma |
| 197 | MOL000923 | ACETIC ACID,BORNYL ESTER | 67.15 | 1.26 | Curcumae rhizoma |
| 198 | MOL000938 | Calarene | 51.55 | 1.82 | Curcumae rhizoma |
| 199 | MOL000939 | (1S,3E,7E,11S)-1,5,5,8-tetramethyl-12-oxabicyclo[9.1.0]dodeca-3,7-diene | 34.37 | 1.58 | Curcumae rhizoma |
| 200 | MOL000940 | Bisdemethoxycurcumin | 77.38 | 0.49 | Curcumae rhizoma |
| 201 | MOL000941 | Zingiberenol | 31.43 | 1.31 | Curcumae rhizoma |
| 202 | MOL000944 | (6R)-2-methyl-6-(4-methylphenyl)hept-2-en-4-one | 40.84 | 1.59 | Curcumae rhizoma |
| 203 | MOL010309 | Cyt | 50.04 | 0.31 | Bolbostemmatis rhizoma |
| 204 | MOL010312 | methyl 4-[2-formyl-5-(methoxymethyl)pyrrol-1-yl]butanoate | 95.34 | 0.51 | Bolbostemmatis rhizoma |
| 205 | MOL010313 | 4-(2-formyl-5-carbooxymethyl-pyrrole-1-yl)-3-phenyl-methyl propanoate | 38.93 | 0.68 | Bolbostemmatis rhizoma |
| 206 | MOL010315 | Beta-sitosterol palmitate | 30.91 | 1.44 | Bolbostemmatis rhizoma |

**Continued Table S1. The ADME properties of 223 potential active ingredients for QYSXC**

| 207 | MOL010316 | Δ7,16,25,26-stigmastatrienol | 46.21 | 1.35 | Bolbostemmatis rhizoma |
| --- | --- | --- | --- | --- | --- |
| 208 | MOL010318 | Δ7,22,25-triene-3-ol | 46.67 | 1.34 | Bolbostemmatis rhizoma |
| 209 | MOL010319 | Δ7,22,25-stigmastatrienol-3-O-nonadecanoate | 40.82 | 1.45 | Bolbostemmatis rhizoma |
| 210 | MOL003518 | Vetol | 53.23 | 0.64 | Bolbostemmatis rhizoma, Strychni semen |
| 211 | MOL001040 | (2R)-5,7-dihydroxy-2-(4-hydroxyphenyl)chroman-4-one | 42.36 | 0.38 | Strychni semen |
| 212 | MOL001476 | (S)-Stylopine | 51.15 | 0.89 | Strychni semen |
| 213 | MOL003019 | Secologanin_qt | 33.33 | -0.1 | Strychni semen |
| 214 | MOL003410 | Ziziphin_qt | 66.95 | 0.49 | Strychni semen |
| 215 | MOL003411 | Icaride A | 48.74 | 0.34 | Strychni semen |
| 216 | MOL003413 | Isostrychnine N-oxide (I) | 35.45 | 0.43 | Strychni semen |
| 217 | MOL003414 | Isostrychnine N-oxide (II) | 37.33 | 0.38 | Strychni semen |
| 218 | MOL003432 | Vomicine | 47.56 | 0.52 | Strychni semen |
| 219 | MOL003433 | Brucine-N-oxide | 49.17 | 0.56 | Strychni semen |
| 220 | MOL003436 | Isobrucine | 33.58 | 0.38 | Strychni semen |
| 221 | MOL003440 | Brucine N-oxide | 52.63 | 0.62 | Strychni semen |
| 222 | MOL003319 | 4-Carboxymethylphenol | 41.89 | 0.05 | Strychni semen |

**Continued Table S1. The ADME properties of 223 potential active ingredients for QYSXC**

| 223 | MOL009314 | Cantleyine | 70.62 | 0.11 | Strychni semen |
| --- | --- | --- | --- | --- | --- |
